# Mammary Tumor Organoid Culture in Non‐Adhesive Alginate for Luminal Mechanics and High‐Throughput Drug Screening

**DOI:** 10.1002/advs.202102418

**Published:** 2021-09-08

**Authors:** Guocheng Fang, Hongxu Lu, Laura Rodriguez de la Fuente, Andrew M. K. Law, Gungun Lin, Dayong Jin, David Gallego‐Ortega

**Affiliations:** ^1^ Institute for Biomedical Materials and Devices School of Mathematical and Physical Sciences University of Technology Sydney Broadway Ultimo Sydney New South Wales 2007 Australia; ^2^ St. Vincent's Clinical School Faculty of Medicine University of New South Wales Sydney Darlinghurst New South Wales 2010 Australia; ^3^ Garvan Institute of Medical Research 384 Victoria Street Darlinghurst New South Wales 2010 Australia; ^4^ UTS‐SUSTech Joint Research Centre for Biomedical Materials and Devices Department of Biomedical Engineering Southern University of Science and Technology Shenzhen Guangdong 518055 China; ^5^ School of Biomedical Engineering Faculty of Engineering University of Technology Sydney Broadway Ultimo Sydney New South Wales 2007 Australia

**Keywords:** alginate, drug screening, high‐throughput, luminal mechanics, mammary tumor organoids, microfluidic droplet

## Abstract

Mammary tumor organoids have become a promising in vitro model for drug screening and personalized medicine. However, the dependency on the basement membrane extract (BME) as the growth matrices limits their comprehensive application. In this work, mouse mammary tumor organoids are established by encapsulating tumor pieces in non‐adhesive alginate. High‐throughput generation of organoids in alginate microbeads is achieved utilizing microfluidic droplet technology. Tumor pieces within the alginate microbeads developed both luminal‐ and solid‐like structures and displayed a high similarity to the original fresh tumor in cellular phenotypes and lineages. The mechanical forces of the luminal organoids in the alginate capsules are analyzed with the theory of the thick‐wall pressure vessel (TWPV) model. The luminal pressure of the organoids increase with the lumen growth and can reach 2 kPa after two weeks’ culture. Finally, the mammary tumor organoids are treated with doxorubicin and latrunculin A to evaluate their application as a drug screening platform. It is found that the drug response is related to the luminal size and pressures of organoids. This high‐throughput culture for mammary tumor organoids may present a promising tool for preclinical drug target validation and personalized medicine.

## Graphical abstract

Mammary tumor organoids culture heavily relies on basement membrane extract hydrogels. Herein, non‐adhesive alginate is found to be a good candidate for the culture of mouse mammary tumor organoids. Alginate microbeads generated by microfluidic droplet technique enhance the organoid's yield and are further used for luminal mechanics and high‐throughput drug screening.

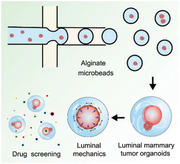

## 1 Introduction

The emergence of mammary tumor organoids as a research tools plays an important role in breast cancer research, pre‐clinical drug screening, and personalized medicine.^[^ ^1^ ^]^ As an in vitro model, mammary tumor organoids are able to recapitulate the pathophysiological microenvironment better than their 2D counterparts. The dimensionality and spatial topology of the 3D environment allows cells to aggregate, interact, and display phenotypes similar to those found in in vivo models.^[^ ^2^ ^]^ Additionally, the dynamic cellular interactions between different cancer‐associated cell types and the extracellular matrix (ECM) regulate tumorigenesis, invasiveness, and mobility.^[^ ^3^ ^]^ Researchers have continued to develop organoids of mammary tumors and other types of cancer to generate in vitro models that can provide a greater predictive value of drug response and treatment efficacy. For example, the Clevers group has built more than a hundred breast tumor organoid lines with cells isolated from tumor tissues.^[^ ^1d^ ^]^ The mammary tumor organoids showed well‐preserved cell lineages and protein expression patterns of the tissue of origin, allowing in vitro drug screens consistent with in vivo xeno‐transplantations and patient response.^[^ ^1d^ ^–^ ^4^ ^]^

To generate biomimetic scaffolds that can simulate the native tumor microenvironment, hydrogels have gained interest as a 3D matrix that can provide physical support, mechanical cues, and binding sites for cells.^[^ ^5^ ^]^ This promotes the formation of highly ordered organoids with hierarchical or luminal structures. The most widely used material for organoid culture is the tumor‐derived basement membrane extract (BME), usually offered as the commercial products Corning Matrigel or Cultrex BME2. BME‐based hydrogels are composed of various ECM proteins, proteoglycans, and some growth factors secreted by Engelbreth–Holm–Swarm murine sarcomas.^[^ ^6^ ^]^ Although BME is easily accessible for researchers as commercial products and is rich in a range of endogenous factors proteins, there are numerous biological drawbacks to using BME as a matrix. First the manufacturing of BME is costly and often consist of batch‐to‐batch variation due to their complicated biochemical components. Second, this variation between batches results in both biological and structural inconsistencies in cell cultures and impacts the reproducibility of experiments.^[^ ^7^ ^]^ Finally, due to their temperature‐sensitive gelation condition, BME is limited and more complex in its application for high‐throughput usage and accurate analysis. Therefore, to address these limitations, synthetic polymers, such as the poly(ethylene glycol)‐based hydrogel^[^ ^8^ ^]^ and naturally‐derived polymers, such as fibrin,^[^ ^8a^ ^]^ were developed for the culture of human intestinal organoids. These synthetic hydrogels allowed researchers to adjust their biochemical components with the addition of chemically‐defined ligands and molecules (such as RGD and laminin‐111), which were supplemented to develop epithelial organoids.^[^ ^9^ ^]^

High throughput culture and analysis of organoids are highly desired to extend the application in drug screening and personalized medicine. Microfluidic and microfabrication technology has been used to increase the throughput of organoid culture. For instance, Brandenberg et al. achieved a high‐throughput culture of gastrointestinal organoids using a microwell array.^[^ ^10^ ^]^ Colon organoids have also been successfully cultured in Matrigel microbeads generated by a droplet method.^[^ ^11^ ^]^ However, the rigorous storage and gelation of Matrigel still remain a challenge in the design and application of microfluidic devices.

Alginate is a natural polymer derived from brown algae, which has been extensively investigated using microfluidic droplet technology due to its mild and fast gelation with divalent cations (e.g., Ca^2+^, Ba^2+^), high biocompatibility, low immunogenicity, and low cost.^[^ ^12^ ^]^ Alginate microbeads can be rapidly generated by microfluidic droplet technology in a high‐throughput manner.^[^ ^13^ ^]^ As alginate is relatively bioinert, the cells embedded in alginate cannot differentiate into ordered structures or typical organoid formation.^[^ ^8^, ^14^ ^]^ To overcome this drawback, alginate can be functionalized via the chemical addition of adhesive and hydrolytic sites.^[^ ^15^ ^]^ An alternative strategy is to use the alginate as an outer scaffolding where the cells can be encapsulated at the core with Matrigel, collagen, or even medium for organoids culture.^[^ ^11^, ^16^ ^]^ In a recent work by M. Capeling et al., non‐adhesive alginate was discovered to be capable of supporting the growth of intestinal organoids by encapsulating the hindgut spheroids with the help of mesenchymal cells.^[^ ^17^ ^]^ However, alginate has not been reported as a 3D support hydrogel for mammary tumor organoid culture.

**Figure 1:**
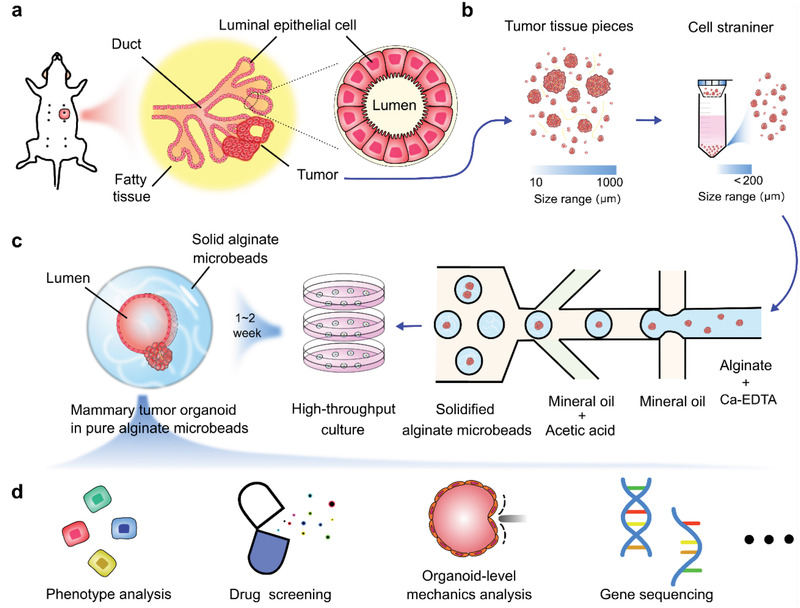
Schematic of high throughput culture of mammary tumor organoids in alginate microbeads. a) Schematic for the structure of mouse mammary tumor. b) Tumor tissues were chopped into pieces and passed through cell strainers. c) High‐throughput generation of alginate microbeads with tumor pieces encapsulated by the microfluidic droplet technique. After 1–2 weeks of culture, mammary tumor organoids formed luminal structures in the alginate microbeads. d) Potential application of the mammary tumor organoids in alginate microbeads.

In this paper, we developed mammary tumor organoids culture from mouse tumor pieces in non‐adhesive alginate microbeads generated with a droplet microfluidic device (**Figure** **1**). The microfluidic chip was fabricated with a homemade soft lithography device. Tumor pieces inside the alginate microbeads formed two types of organoids: luminal and solid types. Flow cytometry analysis indicates that the established organoids showed similar cell compartments to the fresh tumor. The proliferation, luminal structure formation, and key surface markers of the organoids were investigated. Moreover, utilizing the TWPV model, we analyzed mechanical forces from the alginate microbeads to the luminal organoids. Finally, we demonstrated the drug sensitivity analysis of the tumor organoids on a microwell array. We found that the drug response was highly relevant to the size and mechanics of the organoids.

## 2 Results and Discussion

### 2.1 Design Concept of the High‐Throughput Mammary Tumor Organoid Culture in Alginate Microbeads

Within the mammary gland, ducts are composed of a monolayer of luminal epithelial cells surrounded by myoepithelial cells. Uncontrolled growth of cells that line the epithelial acinus and ducts results in the formation of mammary tumors (Figure 1a).^[^ ^18^ ^]^ To test the high‐throughput generation of mammary tumor organoids, the MMTV‐PyMT mouse mammary tumor model was selected. We had chosen the MMTV‐PyMT model as it 1) spontaneously develops multifocal luminal tumors; 2) carcinogenesis have an early onset with 100% penetrance in all mammary glands; and most importantly 3) the primary tumors have a morphology that closely resembles those seen in clinical biopsies.^[^ ^19^ ^]^ Tumors from 12‐ to 14‐week‐old MMTV‐PyMT female mice were harvested and sliced with a Mcllwain tissue chopper until the size of the tumor pieces ranged from 10–1000 µm. The tumor pieces were then filtered by a cell strainer with a pore size of 200 µm, as shown in Figure 1b. We then mixed the tumor pieces (<200 µm) with the alginate/Ca^2+^‐EDTA, which were subsequently encapsulated inside the alginate droplets in the microfluidic chip (Figure 1c). The Ca^2+^ chelated by the EDTA were quickly released upon exposure to an acidic environment to induce the gelation of the alginate microbeads. The alginate microbeads were extracted from the oil and then cultured in tissue culture plates, as shown in Figure 1c. Alginate‐encapsulated tumor pieces were grown several days’ in culture, where half of the organoids were observed to begin developing luminal‐type structures. These luminal structures were found to be the typical morphology of the epithelium organoids, which have also been reported to form in Matrigel culture.^[^ ^20^ ^]^ From this simple method, we achieved a low‐cost and high‐throughput generation of mammary tumor organoids encapsulated in alginate microbeads. These mammary tumor organoids could then be used for high‐throughput drug screening, organoid‐level mechanic analysis, disease mechanism, responses to therapy, clonal evolution, and emergence of drug resistance (Figure 1d). We compared the results encapsulating tumor pieces of different sizes (200–500 µm) in the alginate solution. Tumor pieces below 200 µm were chosen because 1) they can form uniform distribution in alginate and avoid aggregating, 2) receive a good supply of nutrition and oxygen for long‐term culture, 3) effectively avoid channel clogging.

### 2.2 Growth of Mammary Tumor Organoids in Alginate Microbeads

**Figure 2:**
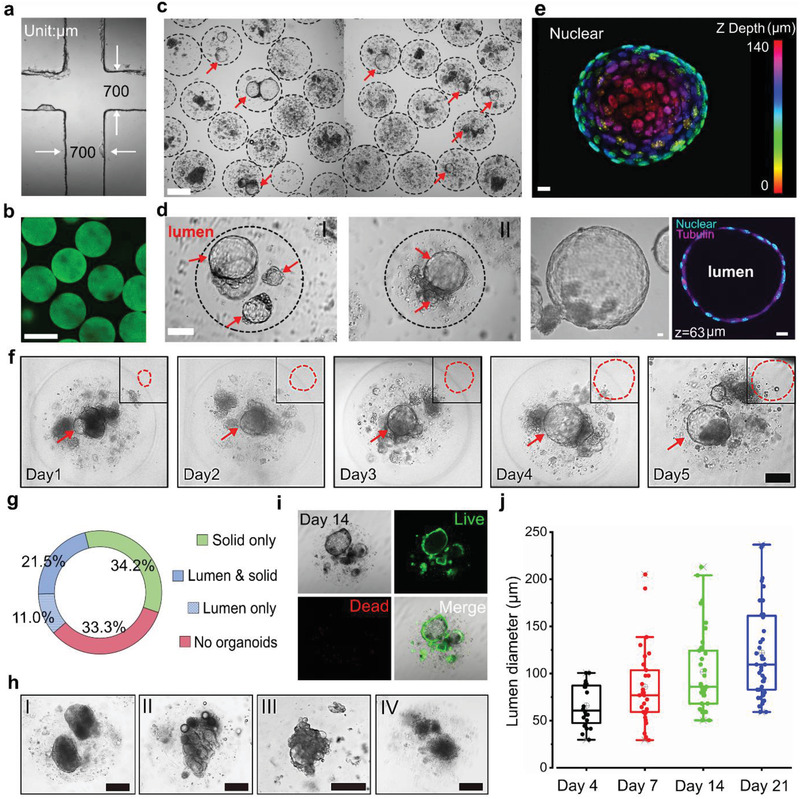
Mammary tumor organoid growth in alginate microbeads. a) Microfluidic chip channel under a 4× objective lens. b) Uniform‐sized fluorescent alginate microbeads. Scale bar: 400 µm. c) High‐throughput generation of mammary tumor organoids in alginate microbeads. Scale bar: 200 µm. d) Enlarged views of the luminal mammary tumor organoids in the microbeads. Scale bar: 100 µm. e) 3D schematic of a 140 µm luminal organoids: nuclear mapping within the *Z*‐axis (upper image) and cross‐sectional view at the *z* = 63 µm (bottom, right). Bright‐field image of the organoid (bottom, left). Scale bar: 10 µm. f) Growth of luminal organoids within five days. Scale bar: 100 µm. g) Percentages of alginate beads containing lumen only organoids, solid only organoids, lumen/solid organoids, and dispersed cells without organoids. h) Solid organoids with smooth boundary (I), blocky structures (II), “grape‐like” structures (III), and discohesive clusters (IV). Scale bar: 100 µm. i) Viability of the mammary tumor organoids in the alginate microbeads at day 14. Scale bar: 200 µm. j) Growth of bulk luminal organoids in the alginate microbeads (mean ± SD, *n* = 20, 24, 33, 40, respectively).

Since our aim was to generate organoids with tumor pieces smaller than 200 µm, we designed the width of the co‐flow channel to be 700 µm to minimize the encapsulation failure and clogging. The channel was fabricated with polydimethylsiloxane (PDMS) based on an SU‐8 mold, manufactured with a homemade soft lithography device. Using a transparent plastic sheet, the photomask with the patterns was printed by an ink‐jet printer for office use (Canon iRADV 4551i), as shown in Figure S1a, Supporting Information. The UV exposure device was made via assembling UV LEDs in an array. The achieved chip channel met the requirement for high‐throughput generation of droplets with several hundred micrometers in diameter, as shown in **Figure** **2**a. Empty alginate microbeads generated within the microfluidics channel displayed uniform sizes, as shown in Figure 2b. However, when alginate was mixed with tumor pieces, the generated microbeads had a range of sizes due to the flow disturbance induced by pieces in the channel, as shown in Figure S2, Supporting Information. In addition, the chip mold can also be made by 3D printers for better control of the channel size.

We observed four types of structures in the microbeads: luminal only organoids, solid only organoids, luminal and solid organoids, and dispersed cells without organized structure. Among them, luminal‐type organoids are of particular interest as they model the hormone receptor‐positive (HR+) breast cancer that constitutes 70% of the diagnosed cases.^[^ ^21^ ^]^ The mammary tumor organoids gradually formed luminal structures in the alginate microbeads. Figure 2c shows the mammary tumor organoids in the microbeads after two weeks’ culture. Notably, the lumens were not formed spontaneously, and the enlarged views of the lumen structures are shown in Figure 2d‐I,II. We stained the nuclei and tubulin to reveal the luminal structure and imaged the organoids in various 3D depths under a laser scanning confocal microscope (Olympus FV‐1200). A 3D nuclei mapping of an organoid with a 140 µm lumen is shown in Figure 2e (upper), including the bright‐field image (Figure 2e (lower left)), and the cross‐section at 63 µm depth of the same luminal organoid (Figure 2e (lower right)). In addition, we tracked the growth of one luminal organoid from day 1 to day 5 (Figure 2f) and observed that the luminal diameter increased from about 60–180 µm within five days at a rate of about 30 µm per day (Figure S3, Supporting Information). The distribution of the organoids in the microbeads on day 14 shows that nearly one‐third (34.2%) and one‐tenth (11.0%) of microbeads contained either solid only or lumen only organoids, respectively; while 21.5% of microbeads contained both solid and lumen organoids. The last third of the microbeads had dispersed cells without organoids (Figure 2g). We then tracked the percentage change during the culture, which indicated that the microbeads containing luminal organoids gradually increased and reached around 40% (Figure S4, Supporting Information).

Solid organoids could also proliferate in the alginate microbeads, as shown in Figure 2c,h. These organoids structures could include smooth boundary(I), blocky structures (II), “grape‐like” structures (III), and discohesive clusters (IV). Mammary tumor organoids with the same structures developed in BME hydrogel have also been previously reported.^[^ ^1d^ ^]^ High cell viability within the encapsulated organoids was maintained throughout the culture on day 7 (Figure S5, Supporting Information) and day 14 (Figure 2i). The average lumen size reached around 60 µm on day 4, 75 µm on day 7 and 85 µm on day 14 and 100 µm on day 21; with a maximum size that could exceed 200 µm. Examples of the luminal mammary tumor organoids released from the microbeads are shown in Figure S6, Supporting Information. In this work, we also embedded MCF‐7 cells inside the alginate microbeads; however, no luminal structure was formed, and only a small number of cell‐loaded microbeads could result in solid multicellular tumor spheroids (Figure S7, Supporting Information). The majority of the cells maintained viability within the microbeads but showed no signs of proliferation. This result indicates that the MCF‐7 cell lines do not maintain a luminal progenitor capacity; thus, they cannot produce the necessary cellular anchorages within the alginate matrix.

Alginate has been widely used as a hydrogel for 3D cell culture and biomedical applications. However, in most cases, the alginate needs to be functionalized with the arginyl glycyl aspartic acid (RGD) peptide to allow cell binding to the scaffold. N. Broguiere et al. found that natural non‐adhesive alginate cannot be used for small intestinal organoids from single pluripotent cells.^[^ ^8a^ ^]^ In contrast, intestinal organoids can be established in non‐adhesive alginate by embedding the hindgut spheroids.^[^ ^17^ ^]^ The author claimed that the importance of co‐culture of mesenchymal cells for organoid formation in non‐adhesive alginate. In our work, the mammary organoids were formed because the tumor pieces were embedded in alginate, which composed of a mixture of various cell types and ECM.

### 2.3 Cell Compartment of Organoids in Alginate Microbeads

**Figure 3:**
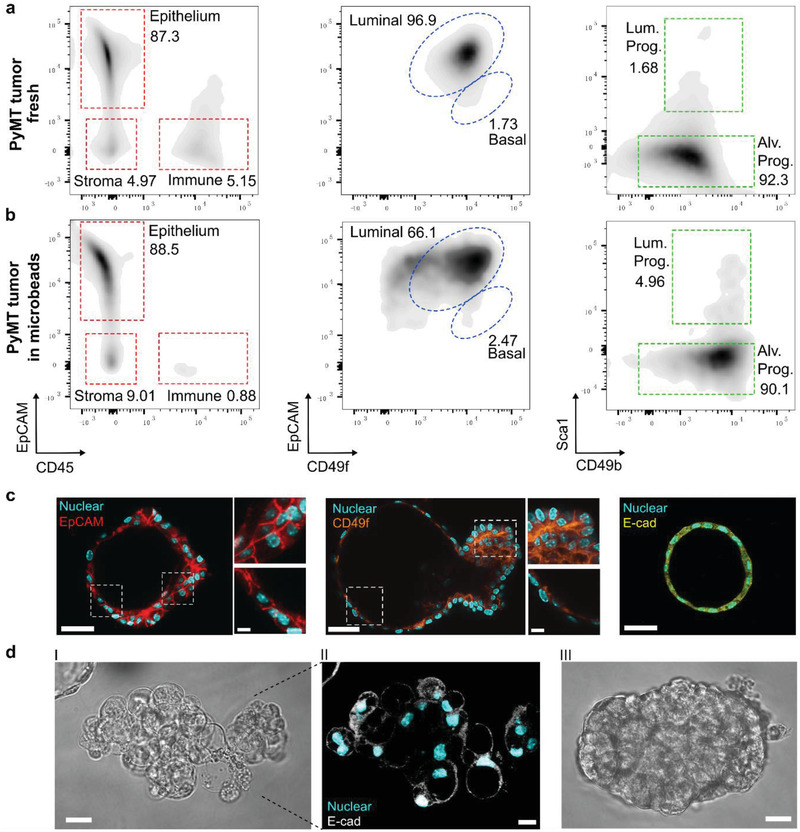
Cell compartments of the organoids. a) Flow cytometry quantification of the cell lineages proportion within fresh PyMT tumors. Major lineages are defined by the expression of EpCAM (Epithelium), CD45 (leukocytes), and double‐negative (stroma). Luminal cells hierarchy is further defined by the expression of CD49f, CD49b, and Sca1. b) Flow cytometry results (as in panel (a)) for PyMT tumor organoids embedded in the alginate microbeads and cultured for two weeks. c) Expression of EpCAM, CD49f, and E‐cad in the luminal organoids. Scale bar: 50 µm, 10 µm (inset). d) Image of the structures with the lumen‐forming potential (I, II) and without the lumen‐forming potential (III). Scale bar: 20 µm.

Mammary gland tissues contain both luminal and basal epithelial lineages. Only the luminal cells consist of HR^+^ and HR^−^ populations, whereas the basal cells only comprise HR^−^ cells.^[^ ^21c^ ^]^ To confirm the cell lineages in the organoids of alginate microbeads, both fresh tumors and organoids collected from the microbeads were digested into single‐cell suspensions and stained with the CD45 and EpCAM/CD49f/Sca1/CD49b antibodies and analyzed by flow cytometry.^[^ ^22^ ^]^ As shown in **Figure** **3**a,b, the percentage of CD45^+^ cells reduced from 5.15% in fresh tumor to 0.88% in alginate microbeads after two‐week culture. This indicates that immune cells encapsulated within the alginate microbeads are unable to survive with the medium composition. In the fresh tumor, luminal cells (EpCAM^+^, CD49f_lo_) and basal cells (EpCAM_lo_, CD49f^hi^) account for 96.9% and 1.73%, respectively. In alginate microbeads, luminal cells and basal cells account for 66.1% and 2.47%, respectively. When looking at the luminal population, HR+ luminal progenitors (Lum. Prog.) accounted for 4.96% in alginate microbeads and (1.68%) in the fresh tumor, while HR‐ alveolar progenitors (Alv. Prog.) had comparable proportions (92.3% in fresh tumors vs 90.1% in microbead cultured tumor organoids). These results indicate that the alginate microbeads could well preserve the luminal cells of the organoids, especially supporting HR^+^ luminal progenitor cells. Immunostaining results for EpCAM, CD49f, and E‐cad of the luminal organoids in the microbeads also confirmed the FACS results (Figure 3c). The formation potential of luminal structures can be distinguished from their deteriorated morphology compared to the solid ones (Figure 3d). FACS results indicate that the organoids developed in the alginate microbeads have a similar epithelial cell composition compared to the fresh tumor. Thus, alginate‐encapsulated organoids resemble mammary tumors and could be a good candidate as an in vitro tumor model.

### 2.4 Immunofluorescence Staining of the Mammary Tumor Organoids

**Figure 4:**
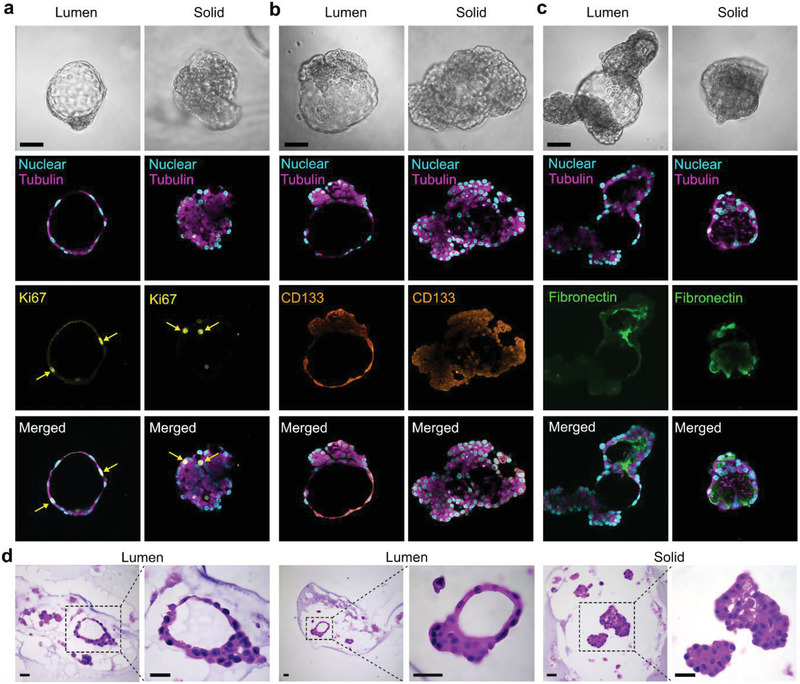
Immunostaining analysis of mammary tumor organoids. a) Ki67 expression as an indicator of cell proliferation in luminal‐type and solid‐type organoids. b) Expression of the CD133 in luminal‐type and solid‐type organoids. c) Fibronectin identification as a measure of ECM in luminal‐type and solid‐type organoids. Scale bar: 100 µm. d) H&E staining of the organoids developed in the alginate microbeads. Scale bar: 50 µm.

The organoids in the microbeads were fixed with paraformaldehyde and stained with antibodies against Ki67, CD133, and fibronectin. Laser scanning confocal microscopy was used to observe the immunofluorescent staining results, as shown in **Figure** **4**. Luminal‐type and solid‐type organoids were observed to have similar Ki67 expression, indicating cell proliferation in the microbeads. We measured the number of the Ki67^+^ cells in the organoids and found there were around 8–10 Ki67^+^ cells in each luminal organoid. CD133 is one of the most commonly used markers of tumor stem cells and is highly expressed in endothelial progenitor cells and mammary glands.^[^ ^23^ ^]^ Both luminal‐type and solid‐type organoids were positively stained with the CD133 antibody, but luminal organoids were found to express a higher level of CD133 compared to solid‐type organoids (Figure 4b). We also stained the organoids for fibronectin, which is a protein that regulates cell adhesion, growth, and migration, and is a critical ECM component for organoid culture.^[^ ^8a^ ^]^ In the luminal‐type organoids, fibronectin was mainly found at the joint between solid and lumen organoids, whereas in the solid‐type organoids, fibronectin mainly distributed at the edge (Figure 4c). Meanwhile, the expression of Ki67, CD133, and fibronectin was very similar to that in the fresh PyMT tumor tissues (Figure S9, Supporting Information). Thus, immunostaining analysis indicated that the organoids cultured in the alginate microbeads could maintain the primary activities. The H&E staining of the luminal and solid organoids showed similar structures to that in the fresh PyMT tumor tissues (Figure 4d; Figure S9, Supporting Information), indicating the reconstitution capacity of the organoids developed in the alginate microbeads.

Here, we found that mouse mammary tumor organoids, especially the luminal type, can be directly derived from tumor pieces in the alginate without any functionalization. Indeed, we found that some alginate microbeads that only contain dispersed single cells could also form the solid‐type and luminal type mammary organoids. This indicates that even single progenitor cells in the alginate can grow into mammary organoids, given that ECM and mesenchymal cell may also be co‐cultured. We also found that not all the organoids developed a lumen at the same time; some organoids formed a luminal‐like structure after one day of culture, while others appeared after 3–4 days of culture. This highlights that the luminal progenitor activity in the alginate microbeads might be under different situations (perhaps different cell cycles), which results in the luminal formation at different times.

### 2.5 Mechanics of Luminal Organoids Measured in Alginate Microbeads

Epithelial tissues, endowed with physiological functions, mainly rely on the intricate structures that usually consist of lumens.^[^ ^24^ ^]^ The disorder or loss of the lumens is usually related to development defects, inflammatory reactions, and cancer.^[^ ^25^ ^]^ The fluid‐filled lumens, due to their incompressibility, can transmit the hydraulic force to coordinate the cellular functions at the scale of a whole organ or even embryo. The stress exerted by luminal mechanics can change tissue geometry and tension distribution.^[^ ^26^ ^]^ These changes in mechanics and morphology could directly guide the cell behavior and fate, often via mechanotransduction.^[^ ^27^ ^]^ Therefore, luminal mechanics play an essential role in tissue morphogenesis. For instance, during lung organogenesis, the sustained stretch applied on undifferentiated pulmonary mesenchymal cells by luminal fluidic pressure can active the serum response factor, leading to smooth muscle differentiation. The expansion value calculated by the luminal pressure could help the therapy of lung hypoplasia, where the pressure is artificially increased.^[^ ^28^ ^]^ The luminal pressure could influence the size of lymph vessels via guiding the differentiation and morphology of the lymphatic endothelial cells. The mechanics help to understand the development of the lymphatic system since the embryo stage.^[^ ^29^ ^]^

Organoid lumen formation arises from the interplay of mechanical and biochemical signaling.^[^ ^30^ ^]^ Measuring biomechanics,^[^ ^31^ ^]^ especially the luminal mechanics,^[^ ^26^ ^]^ has been the subject of intense scholarly debate. Various methods have been established to measure the luminal pressure, such as the micropressure probe, gel deformation assay, pressure sensors, deformable beads, traction microscopy, and atomic force microscopy.^[^ ^32^ ^]^ It is worth mentioning that gel deformation assay is a non‐invasive and direct method, enabling the relatively wide pressure range. For instance, alginate capsules generated by electrospray could sense the pressure loaded by the epithelium lumen.^[^ ^25b^ ^]^ However, this method could monitor the luminal pressure only when the lumen size grows comparable to the capsule size. Furthermore, it requires a thin wall of the capsule, challenging the fabrication technique and skills. Here, we established the mechanical analysis of the luminal organoid in the alginate microbeads based on the theory of TWPV model, which could calculate the mechanics throughout the development of the organoids.

**Figure 5:**
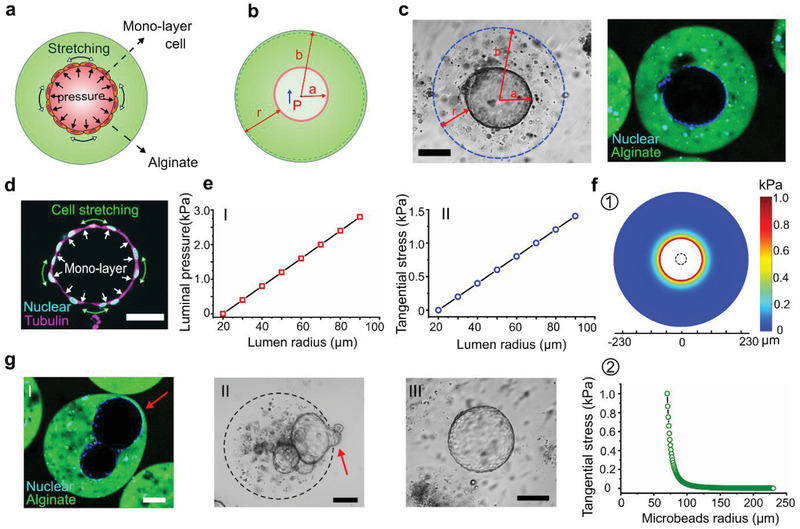
Organoid mechanical analysis in microbeads based on the TWPV theory. a) Luminal pressure and stretching stress of the luminal organoids in the alginate microbeads. b) Luminal expansion model based on TWPV theory and its main parameters. c) Bright‐field image of an organoid at the center of an alginate microbead and its expansion in fluorescent alginate microbeads. d) Fluorescence image of the organoids with monolayer cell connections. e) Lumen radius versus the luminal pressure (I) and tangential stress (II). f) Stress distribution on the microbeads when the luminal pressure is 2 kPa (①), and its distribution along the radius (II). g) Large organoids extruded from the alginate microbeads(②); luminal pressure leads to the hump exposed to the outside (II); large organoid released from the alginate microbeads (III). Scale bar: 100 µm.

As shown in **Figure** **5**a, the luminal organoids in the alginate microbeads face the radial luminal pressure and the tangential stretching stress, where the organoid is supposed to be at the center of the microbeads. The parameters are set as shown in Figure 5b: *a* is the inner radius, *b* is the outer radius, *r* is the wall thickness, and *P* is the luminal pressure. Since alginate is a linear elastic material,^[^ ^33^ ^]^ we can use the TWPV model (*b *< 10*r*) to calculate the pressure. In the theory of the TWPV model, the outer radius would not change much with the expansion of the lumen, which was demonstrated by our observation during the culture. Bright‐field imaging of the luminal organoid at the center of the microbeads supports the TWPV theory (Figure 5c). We then synthesized fluorescent alginate to confirm the expansion of the lumen, where we were able to verify that no alginate had entered inside the lumen of organoids. Moreover, the fluorescent alginate allows the observation of deformation more prominently. According to the TWPV model, the luminal pressure can begiven by^[^ ^34^ ^]^

(1) $$P=\frac{-4G\;\left(b^{3}-a^{3}\right)}{2ma^{4}+ab^{3}}\Delta u$$

where *m* = (1 − 2*v*)/(1 + *v*), *v* is the Poisson's ratio, *G* is the shear modulus of the material, and Δ*u* is the expansion displacement. The tangential stress along the radius can be given by^[^ ^34^ ^]^

(2) $$\sigma_{t}=\frac{a^{3}P}{2\left(b^{3}-a^{3}\right)}\left(2+\frac{b^{3}}{x^{3}}\right)$$

where *x* is the radius of interest between *a* and *b*.

Here, the initial radius *a* of the lumen is set as 20 µm, the initial outer radius *b* of the microbeads is set as 230 µm, and the shear modulus of the alginate is 203 Pa according to the literature.^[^ ^35^ ^]^ The concentration of the alginate started at 1 wt%. However, when mixing the alginate with the cell pellet, a small portion of the medium was mixed, leading to the final concentration of alginate lower than 1 wt%. Thus, we referred to the shear modulus of around 0.7% alginate. According to Equation (1), the relationship between luminal pressure and lumen radius is shown in Figure 5e‐I. We could see that when the luminal diameter reaches 150 µm, the luminal pressure is about 2 kPa. This value is lower than 3 kPa, which is applied by solid tumor spheroids in the alginate capsule.^[^ ^33^ ^]^ Interestingly, it is slightly higher than the pressure generated by mammalian embryos at 100 µm diameter (1.5 ± 0.3 kPa).^[^ ^36^ ^]^ The result also shows that the luminal pressure of these organoids is much higher than the pressure of epithelial lumen cultured in scaffold‐free medium (0.12 kPa).^[^ ^32b^ ^]^ According to Equation (2), the relationship between tangential stretching stress and lumen radius is shown in Figure 5e‐II. We found that when the lumen diameter reaches 150 µm, the tangential stretching stress reaches 1.1 kPa. Previous reports suggest that the cell monolayer can sustain this stress with approximately 20% extension,^[^ ^25a^ ^]^ which supported our observation. Fluorescence imaging of the lumen showed the monolayer cell connections and the extended‐like performance (Figure 5d).

We further characterized the stress distribution on the deformed alginate microbeads along the radius when the luminal pressure is 2 kPa (Figure 5f). The stress has a significant decrease along the radius, which indicates that the whole size of the alginate microbeads would not change much in radius. We were also able to identify some larger organoids that extrude the alginate microbeads during the culture (Figure 5g‐I). Some of the organoids with the surface exposed to the outside of the alginate can form a “hump,” which is caused by high luminal pressure (Figure 5g‐II). Organoids that were too large were found to penetrate through the alginate microbead, destroying the encapsulation (Figure 5g‐III). These exposed organoids attached to the cell culture dish could sustain the lumen structure for around one day before collapsing for further spokewise growth (Figure S8, Supporting Information). Here we found that the TWPV model based on the alginate microbeads offers a simple and effective method to analyze the luminal mechanics of the organoids.

### 2.6 Cellular Response and Luminal Pressure Relationship to Drug Treatment

**Figure 6:**
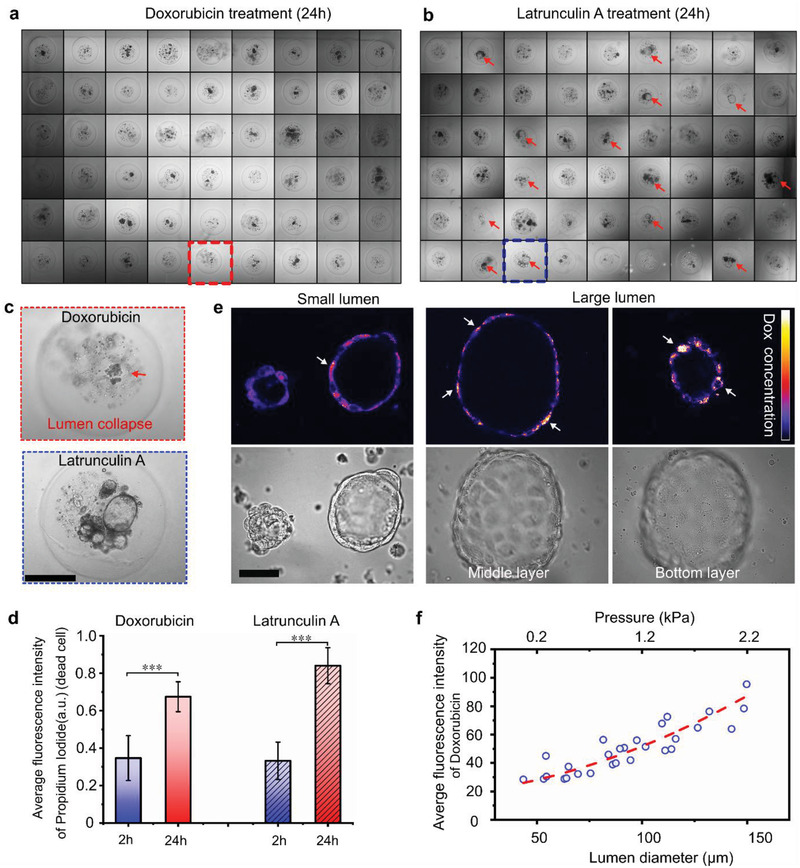
Cell response of mammary tumor organoids in drug screening. a) Organoids in the agarose array after 24 h treatment of doxorubicin: no lumen could be observed. b) Organoids in the agarose array after 24 h treatment of latrunculin A: lumen could be observed. Red arrows indicate the lumens. c) Enlarged view of the lumen after the drug treatment. Scale bar: 200 µm. d) The fluorescence intensity of dead cells after the drug treatment. The values were normalized against the highest intensity (mean ± SD, *n* = 10, ***p < 0.001). e) Fluorescence images of doxorubicin uptake in different‐size organoids. Scale bar: 50 µm. f) The correlation of the doxorubicin uptake, organoid size, and luminal pressure.

Drug screening requires high‐throughput culture and analysis of organoids. We loaded the microbeads into an agarose microwell array and treated the organoids with doxorubicin, latrunculin A or vehicle controls, respectively. Doxorubicin and latrunculin A are two standard anti‐cancer drugs in the treatment of breast cancer. The agarose microwell array (9 × 9) was fabricated via a MicroTissues micro‐mold (Sigma‐Aldrich). The microwell array was placed in a six‐well plate and immersed in the culture medium. Each microwell held only one alginate microbead. According to the percentage of microbeads containing only lumens, luminal and solid organoids, and only solid organoids are 11%, 21.5%, and 34.2%, respectively (Figure 2g), there should be eight microwells containing only lumens, 17 microwells containing both luminal and solid organoids, 27 microwells containing only solid organoids. Here we focus on the cellular response of the luminal organoids. After 24 h, doxorubicin treatment resulted in a collapse and disappearance of the luminal structures within the microbeads (**Figure** **6**a).

On the contrary, after 24 h treatment of latrunculin A, the lumens in microbeads still existed, as shown in Figure 6b,c. The different response was presumably caused by the different mechanisms of action of the two drugs. Doxorubicin interacts with DNA by intercalation and inhibition of macromolecular biosynthesis. This inhibits the progression of topoisomerase II, an enzyme that relaxes supercoils in DNA for transcription. It may also increase quinone type free radical production, which may destroy the structure of the lumen structure. On the other, Latrunculin A affects polymerization of actin and the prevention of polymerizing of the actin filaments causes reversible changes in the morphology of mammalian cells.

We assessed the viability of the 14‐day‐old organoids with propidium iodide and/or calcein‐AM after the drug treatment. Despite the different anti‐tumor mechanisms, both drugs produced widely spread cell death. The doxorubicin fluorescence overlaps with that of calcein‐AM. Thus, the organoids treated with doxorubicin were only analyzed with propidium iodide. The averaged fluorescence intensity of propidium iodide was used to evaluate dead cells after 2 and 24 h (Figure 6d; Figure S10, Supporting Information). After 2 h treatment, the average intensity of dead cells was similar between the two treated groups. After 24 h, the intensity of the latrunculin A group was slightly higher than that of the doxorubicin group. We also compared the drug uptake efficiency of the different‐sized organoids. After 2 h treatment, the fluorescence of doxorubicin was examined under confocal microscopy. As shown in Figure 6e,f, a noticeable trend was observed: organoids with a larger size and higher luminal pressure showed higher doxorubicin uptake. Consistent with previous research,^[^ ^37^ ^]^ large organoids sustained strong mechanical pressure, leading to more considerable stretching and deformation of the cells for higher cellular uptake of drugs. To verify whether the different diffusion distances can lead to uniform concentration in microbeads, we tested the diffusion process of doxorubicin, Rhodamine B and IgG proteins. As shown in Figures S11–S13, Supporting Information, these compounds can fast diffuse into the alginate microbeads and lead to a uniform distribution. So, in conclusion, our results indicate that the mammary tumor organoids in the alginate microbeads show promising potentials in drug screening.

In this work, the organoids were derived from the MMTV PyMT mouse mammary tumor. Therefore, we hypothesize that the use of breast cancer patient tumor tissues could be used to form organoids in alginate microbeads. This, in turn, may provide a clinically relevant drug screening platform for breast cancer. We envision alginate microbead encapsulation of other types of tumor tissues will expand the application of this platform.

## 3 Conclusions

Mouse mammary tumor organoids, especially the luminal type, were developed in non‐adhesive alginate microbeads generated by a microfluidic droplet device. Flow cytometry, live/dead staining, and immunofluorescent staining results indicated that the established organoids maintained their phenotypes and similar cell epithelial compartments to the fresh tumor. A TWPV model based on the deformation of the alginate microbeads was established to analyze the mechanics of the luminal organoids. The organoids in alginate microbeads were used for the drug screen. The organoids showed a remarkably different response to doxorubicin and latrunculin A. Organoids size and luminal pressure influenced the drug uptake. This platform offers a versatile and low‐cost strategy for luminal‐mechanics analysis, high‐throughput generation, and drug screening of mammary tumor organoids. Together, these findings may pave the way for alginate as a promising material for high throughput tumor organoid culture.

## 4 Experimental Section

### Microfluidic Chip Fabrication and Process

Due to the simple structure and the relatively large width (a few hundred micrometers), the chip could be fabricated by 3D printing or homemade soft lithography. In this paper, the latter method is shown. First, the mask was printed on a transparent plastic sheet via a commercial printer (iRADV 4551i Canon). The pattern was drawn in the Adobe Illustrator CS6. Two overlapped layers were used as the mask to reduce the roughness of the edge and enhance the transmission, as shown in Figure S1, Supporting Information. Then, the SU‐8 2150 photoresist was spin‐coated on the 4‐in. silica wafer with a 550 µm thickness. Next, the photoresist, covered with the mask, was exposed under a UV LED for 2 min, which was followed by the standard post bake, developing and hard bake process. After the fabrication of the SU‐8 mold, the PDMS was mixed with the curing agent in a ratio of 10:1 and then poured on the mold. Finally, the peeled‐off PDMS was bonded with a glass slide after air plasma treatment.

### Mouse Tumor Tissue Process

The mouse experiments were performed at the Garvan Institute of Medical Research under the approval of the St. Vincent's Campus Animal Ethics Committee (AEC #19/02). MMTV‐PyMT mice were induced to generate tumor tissues. The mouse PyMT tumor tissue was collected after four weeks and chopped into small pieces in the culture medium. The pieces were passed through pluriStrainer cell strainers. Pore sizes ranging from 100 to 500 µm were compared, and only the 200 µm strainers were used for further experiment.

### Alginate Microbeads Generation and Collection

Sodium alginate (Sigma‐Aldrich) was dissolved in phosphate‐buffered saline (PBS) with a concentration of 2 wt% under the ultrasound. The calcium‐EDTA was generated by mixing the disodium‐EDTA solution (100 × 10^−3^ m) and the calcium solution (100 × 10^−3^ m) with a ratio of 1:1. The pH of the calcium‐EDTA was finally adjusted to around 7.4 by adding sodium hydroxide solution. Then the sodium alginate was mixed with the calcium‐EDTA solution with an equal ratio. The final concentration of the alginate was 1 wt%. Under this condition, the calcium irons were chelated inside the EDTA and would not gelate with the alginate chains. Span 80 (Sigma‐Aldrich) was added inside the mineral oil (Sigma‐Aldrich) at a concentration of 3.5 vol% for the second inlet. Mineral oil mixed with 3.5 vol% span80 and 0.08 vol% acetic acid was prepared for the third inlet. The channel surface was treated as hydrophobic by 1 wt% 1H,1H,2H,2H‐perfluorododecyltrichloro in isopropanol.

The tissue pieces less than 200 µm were centrifuged, and the pellet was mixed with the alginate‐calcium‐EDTA solution. Due to some medium remained at the bottom, the final concentration of the alginate should be lower than 1 wt%. The alginate, mineral oil, and mineral oil with acetic acid were introduced into the chip respectively via the syringe pump. The droplets generated at the junction would gelate when exposed to the acidic mineral oil because the acid in the mineral oil would release the calcium ions from the EDTA. After that, the alginate microbeads were collected in the tube where some culture medium was at the bottom, and some mineral oil with acetic acid was at the top. The alginate microbeads would naturally settle from the mineral oil to the medium. Finally, the microbeads were transferred to the cell culture dish for further culture.

### Mammary Tumor Organoids Culture

The alginate microbeads were cultured in the medium in six‐well cell culture plates, as shown in Figure S14a, Supporting Information. The medium contained the DMEM medium with 10% fetal bovine serum (FBS), 5 µg mL^−1^ insulin, 1% HEPES, 1% glutamine, 10 ng mL^−1^ human epithelial growth factor (hEGF), and 10 ng mL^−1^ cholera toxin. The medium was replaced every two days.

### Immunofluorescent and H&E Staining

The 2‐week‐old organoids were used for the immunofluorescent staining. First, the medium was removed from the six‐well plate. Then 2 mL Tris Acetate‐EDTA buffer was added to degrade the alginate gradually. After 10 min, the released organoids were collected to a 96‐well plate. The organoids were fixed with 4% paraformaldehyde for 20 min. After washing three times by PBS, 100 µL 0.5% Triton X‐100 was added to each well and incubates at room temperature for 1 h. After washing three times with PBS, the organoids were then blocked with 5% bovine serum albumin (BSA)/PBS solution at 37 °C for 1 h. Next, the anti Ki67, CD133, and fibronectin antibodies (Sigma‐Aldrich) (1:200, 1:50, 1:200, dilution in 0.1% BSA/PBS) were added to the well respectively, and incubated at 37 °C for 1 h. After washing thrice with PBS, the cells were immersed in the Cy3‐labelled second antibody solution (1:500 dilution in 0.1% BSA/PBS) at 37 °C for 1 h. Finally, after washing thrice with PBS, the Hoechst 33342 (1:1000) and Tubulin Tracker Deep Red (1:1000) were applied and incubated for 10 min, before the observation under confocal microscopy. The fresh tumors were fixed in 10% neutral buffered formalin, embedded in paraffin, sectioned, and deparaffinized before staining. The slides were treated with 10 mm citrate buffer at 95 °C for 10 min for antigen retrieval. H&E, immunohistochemical, and immunofluorescent staining were used to characterize the samples.

### Flow Cytometry

Fresh tumor tissues were chopped into small pieces and then enzymatically dissociated in DMEM/F12 (1:1) supplemented with 2 mg mL^−1^ collagenase and 200 U mL^−1^ hyaluronidase. After dissociation, red blood cells were lysed in ammonium chloride and processed to a single cell suspension by sequential digestion with 0.25% Trypsin, 5 mg mL^−1^ dispase, and 1 mg mL^−1^ DNase, and filtered through a cell strainer. Organoids in the microbeads were collected and treated with Tris Acetate‐EDTA buffer to remove the alginate and then dissociated into single cells with TrypLE. Single‐cell suspensions were then incubated with primary antibodies, incubated with 4′,6‐diamidino‐2‐phenylindole (DAPI), and analyzed by flow cytometry using a FACSAria II.

### Synthesis of Fluorescent Alginate

The synthesis of the fluorescent alginate followed the work.^[^ ^38^ ^]^ First, alginate was dissolved in PBS (PH 7.2‐7.4, Sigma‐Aldrich) to give approximately 90 mm carboxylic groups. Then, EDC (1‐Ethyl‐3‐(3‐dimethylaminopropyl) carbodiimide hydrochloride) and sulfo‐NHS (*N*‐hydroxysulfosuccinimide sodium salt) were added to 9 mm of each. The solution was kept at room temperature with stirring for 2 h. Next, 0.45 mm fluoresceinamine was added. The reaction was stirred at room temperature for 18 h. Then, the solution was transferred to dialysis membranes (12 000–14 000) and dialyzed against ion‐free water overnight at 4 °C. Then the solution was dialyzed in 1 m NaCl for 24 h until the water lost yellow color (5 shifts). Finally, the solution was freeze‐dried protected from light.

### Cell Viability and Drug Treatment in the Agarose Array

The 2‐week‐old organoids were used for drug treatment. The microwell agarose array was generated following the previous work^[^ ^39^ ^]^ or the commercial microwell mold (Z764019, Sigma‐Aldrich), as shown in Figure S14b, Supporting Information. Before using the microwell plate, the agarose plate was immersed in DMEM for at least 2 h. Then the microbeads were added to the microwell via a pipette. Next, the medium was removed, and then 2 mL fresh medium with 3.5 µm doxorubicin or 1 µm latrunculin A was added, separately. In the control group, 2 mL fresh medium with the same volume of PBS was added. The medium was changed every 2 days. The viability was measured by the propidium iodide and/or calcein‐AM straining. Then the microbeads were imaged under a confocal microscope.

### Statistical Analysis

The data were presented by means ± standard deviation (SD). A two‐tailed *t‐*test was used to reveal the statistical difference in Figure 6d with Origin 8.0. A *p*‐value less than 0.05 was considered as significant difference (*p < *0.05).

## Conflict of Interest

The authors declare no conflict of interest.

## Supporting information

Supporting Information

## Data Availability

The data that support the findings of this study are available from the corresponding author upon reasonable request.
