# Supplementary material for: Mammary Tumor Organoid Culture in Non‐Adhesive Alginate for Luminal Mechanics and High‐Throughput Drug Screening
Source: Adv Sci (Weinh). 2021 Sep 8;8(21):2102418. doi: 10.1002/advs.202102418 (PMC8564453; doi:10.1002/advs.202102418)
Supplement: Supplementary file 1 — Supporting Information [file ADVS-8-2102418-s001.pdf]

© 2021 Wiley-VCH GmbH

ADVANCED SCIENCE
Open Access

Supporting Information

for *Adv. Sci.,* DOI: 10.1002/advs.202102418

Mammary tumor organoid culture in non-adhesive alginate for luminal mechanics and high-throughput drug screening

Guocheng Fang, Hongxu Lu, Laura Rodriguez de la Fuente, Andrew M.K Law, Gungun Lin, Dayong Jin*,and David Gallego-Ortega**

Supporting Information

Mammary tumor organoid culture in non-adhesive alginate for luminal mechanics and high-throughput drug screening

Guocheng Fang, Hongxu Lu, Laura Rodriguez de la Fuente, Andrew M.K Law, Gungun Lin, Dayong Jin*, David Gallego-Ortega**

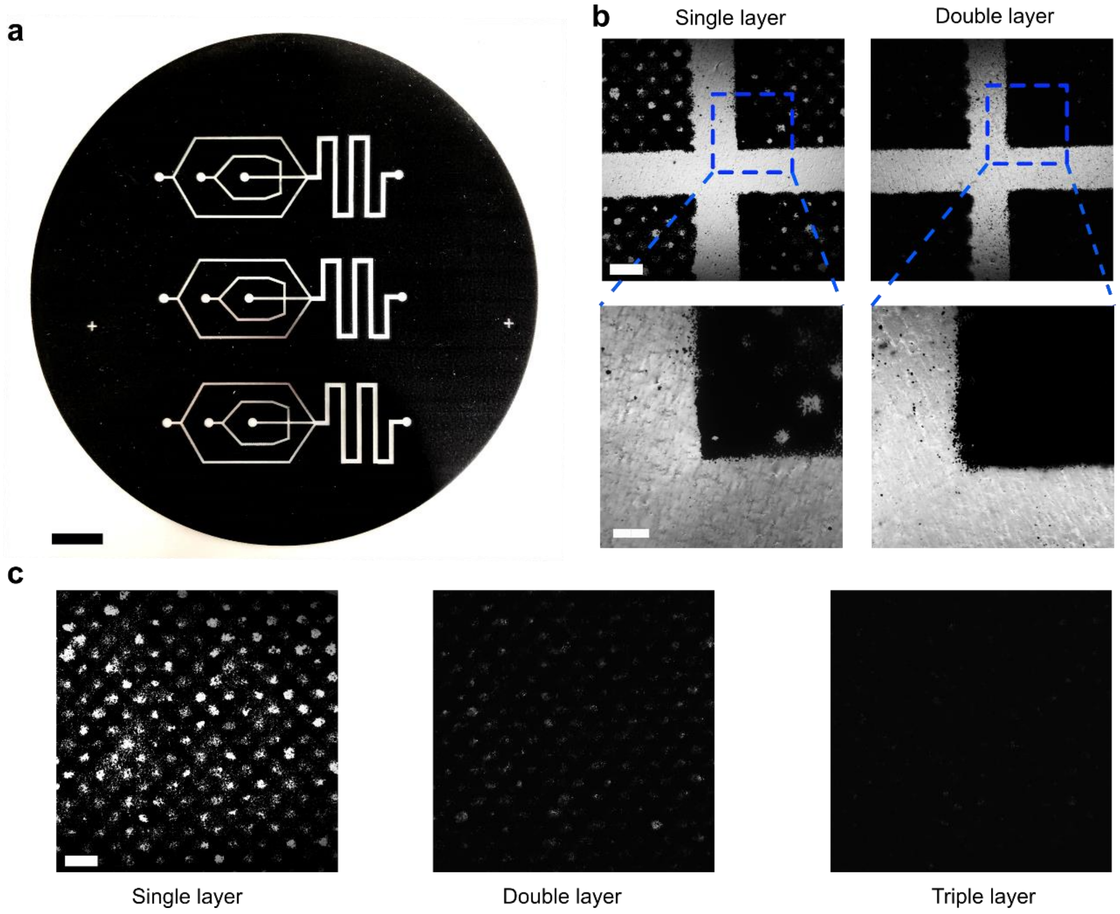

Figure S1. (a) Double-layer plastic mask fabricated by a commercial ink-jet printer. Scale bar: 1cm. (b) Bright-field image of the mask under a microscope with single and double layers, respectively. Scale bar: 500µm. Scale bar of the enlarged view: 200µm. (c) Transparency of the mask with single, double and triple layers. Scale bar: 500µm.

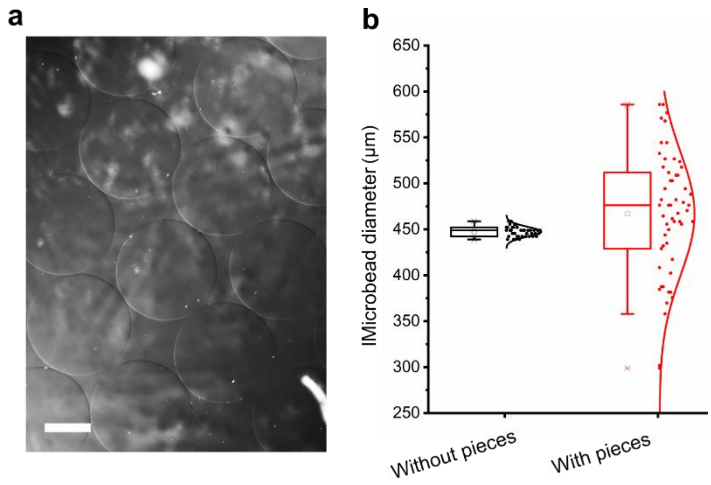

Figure S2. (a) Uniform-sized unloaded alginate microbeads. Scale bar: 200 μm. (b) Size distribution of the alginate beads without/with pieces inside. The pieces in the channel disturbed the flow, leading to the relative un-uniformed size of the beads.

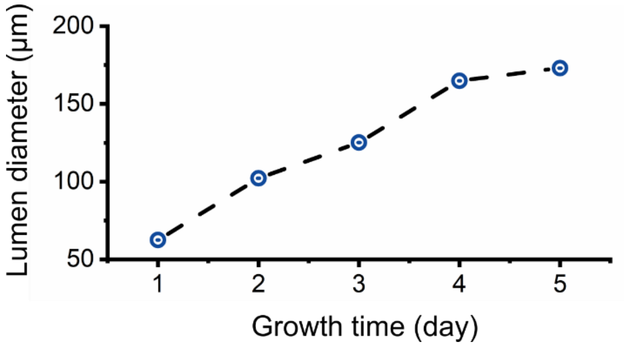

Figure S3. Lumen diameter increasing during the culture.

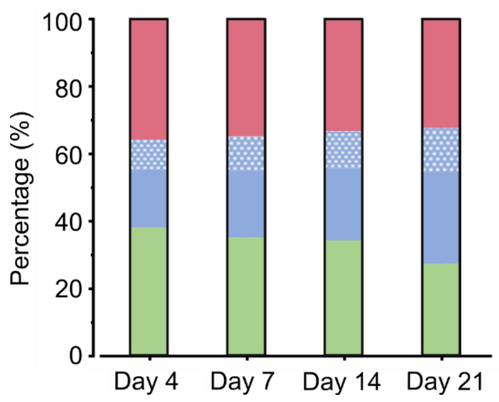

Figure S4. Percentage of microbeads containing solid only organoids, lumen only organoids, solid&lumen organoids, and no organoids (dispersed cells) at day 4, day 7, day 14 and day 21, respectively.

As shown in Figure S4, the percentage of microbeads containing only solid organoids decreased from 38.1% on day 4 to 27.3% on day 21. The percentages of microbeads containing both lumen and solid organoids grew from 17.1% on day 4 to 27.3% on day 21. The percentage of microbeads containing only lumen organoids varied from 8.6% on day 4 to 13.1% on day 21. The percentage of microbeads containing no organoids (dispersed cells) decreased from 36.2% to 32.3%. It indicates that more lumen organoids gradually formed during the culture, which is why the percentage of microbeads containing only solid organoids decreased.

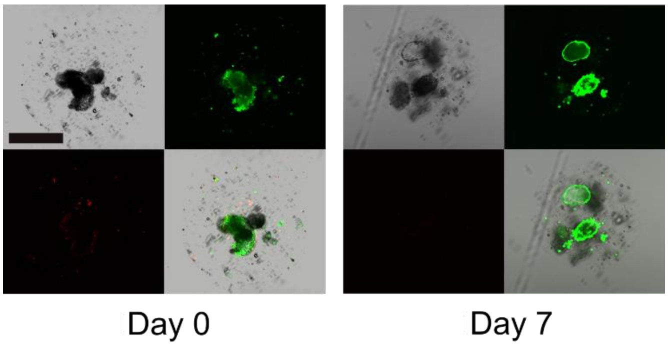

Figure S5. Viability of the mammary tumor organoids in alginate microbeads at day 0 and day7. Scale bar: 200 μm.

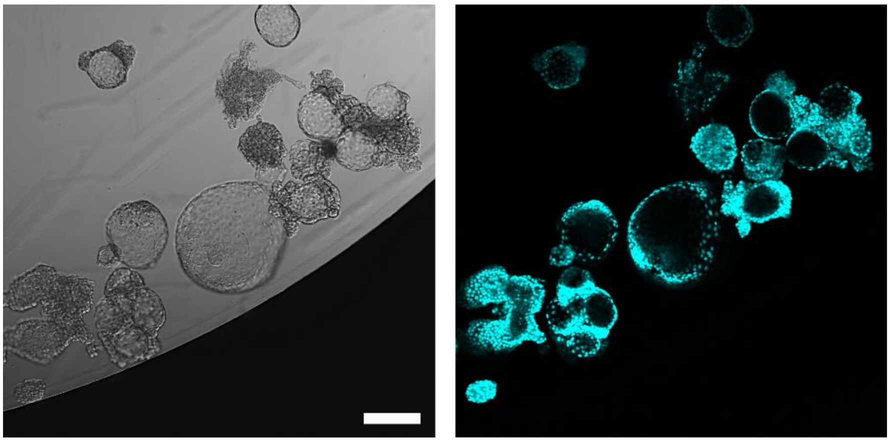

Figure S6. Luminal mammary tumor organoids released from the alginate microbeads (cyan: nuclei). Scale bar: 100 μm.

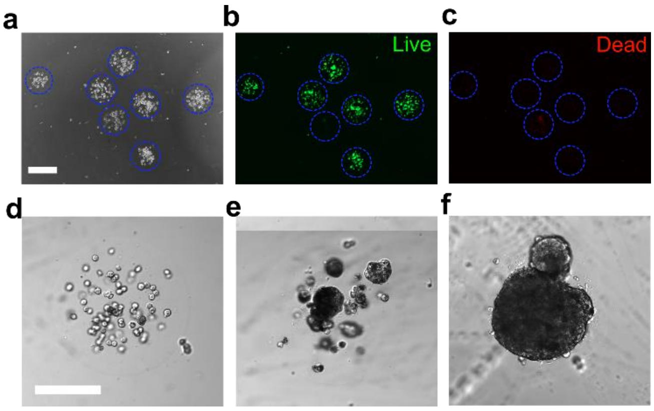

Figure S7. (a) Bright-field image of the single MCF-7 cells encapsulated in the alginate microbeads. (b)&(c) Cell viability in the microbeads staining by calcein-AM and propidium iodide. Scale bar: 200 µm. (d) After 2-week culture, most cells remain alive but no proliferation. (e) Small solid tumor formed in alginate microbeads. (f) Large solid tumor spheroid formed in about alginate microbeads. Scale bar: 100 µm.

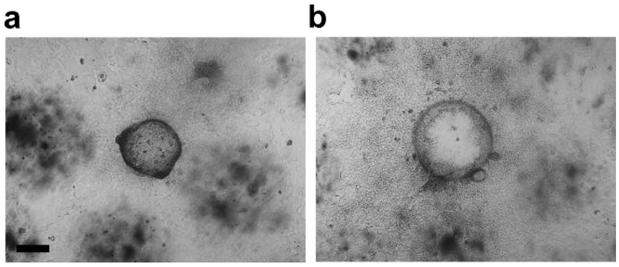

Figure S8. (a) Organoid released from alginate microbeads attached to the dish. (b) The released organoid collapsed and grew into a spokewise shape. Scale bar:100µm.

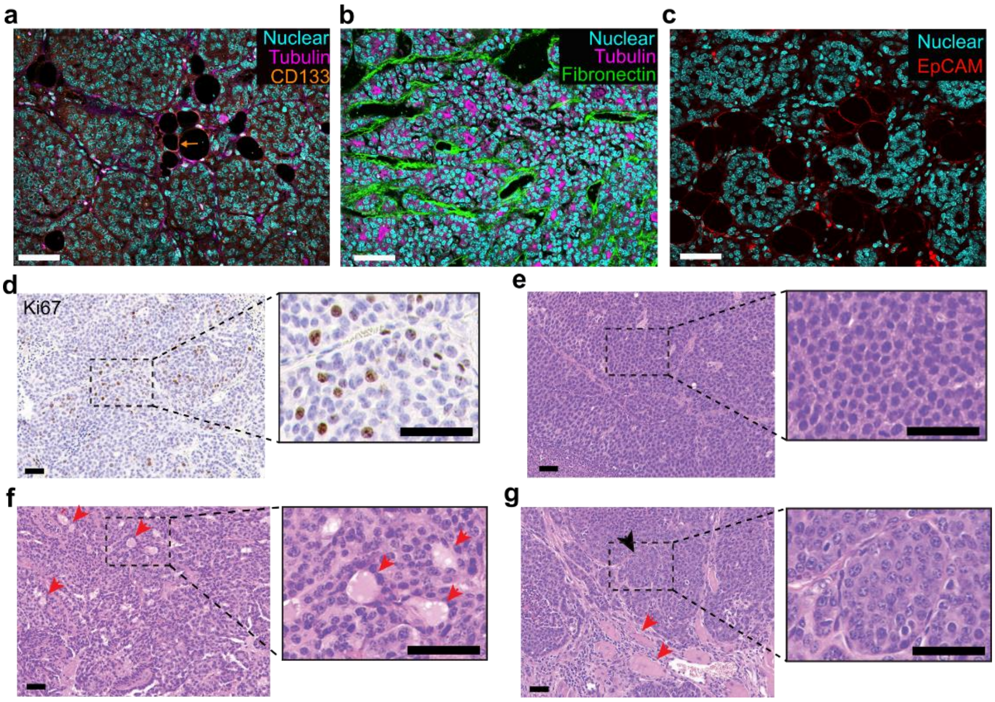

Figure S9 Immunofluorescent and H&E staining images of the fresh PyMT tumor tissues. (a) Expression of CD133, (b) expression of fibronectin, (c) expression of EpCAM, (d) expression of Ki67.(e-g). Immunohistochemistry of the fresh PyMT tumor tissues. Sable bar: 50 µm.

We found that the expression of CD133, fibronectin, EpCAM and Ki67 in alginate organoids was similar to the fresh PyMT tumor. In the fresh tumor, CD133 was expressed more in the luminal area, identical to the organoids. In the fresh tumor, the fibronectin staining was more intensive at the joint of lumen and solid area, similar to the organoids. The number of Ki67+ cells was almost the same.

MMTV-PyMT tumors were presented with heterogeneous histology, partly due to the development of multiple tumor foci with different progression stages in each mammary gland. Most tumors from 12-week-old mice have progressed to late carcinoma, characterized by a continuous sheet of epithelial cancer cells (**Figure S9e**). However, several foci still show adenoma/early carcinoma morphology with discrete acini of ducts filled by cancer cells surrounded by aspects of basal membrane and connective tissue (**Figure S9 f-g**, black arrow). Other areas still remained scattered by adenoma-like liquid-filled cysts (Figure R6f-g, red arrow). Similar structures can be found in the organoids developed in the alginate microbeads (**Figure S9 f-g**, red arrow).

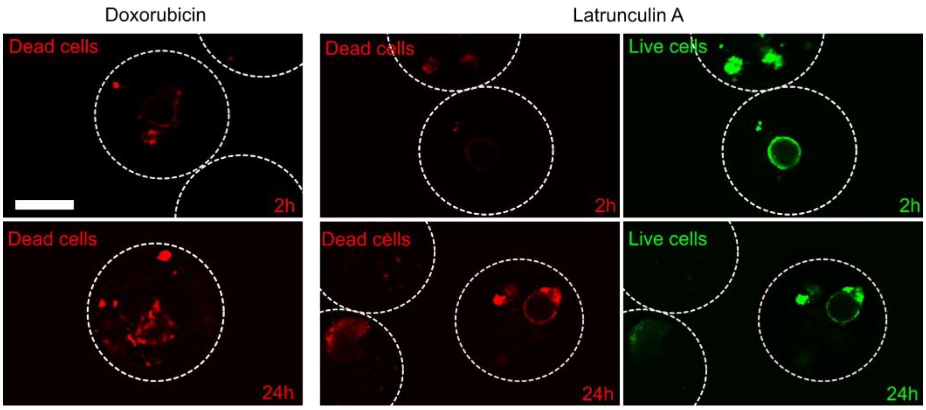

Figure S10. Live and dead staining images of the organoids in alginate microbeads after drug treatment. Scale bar: 200μm.

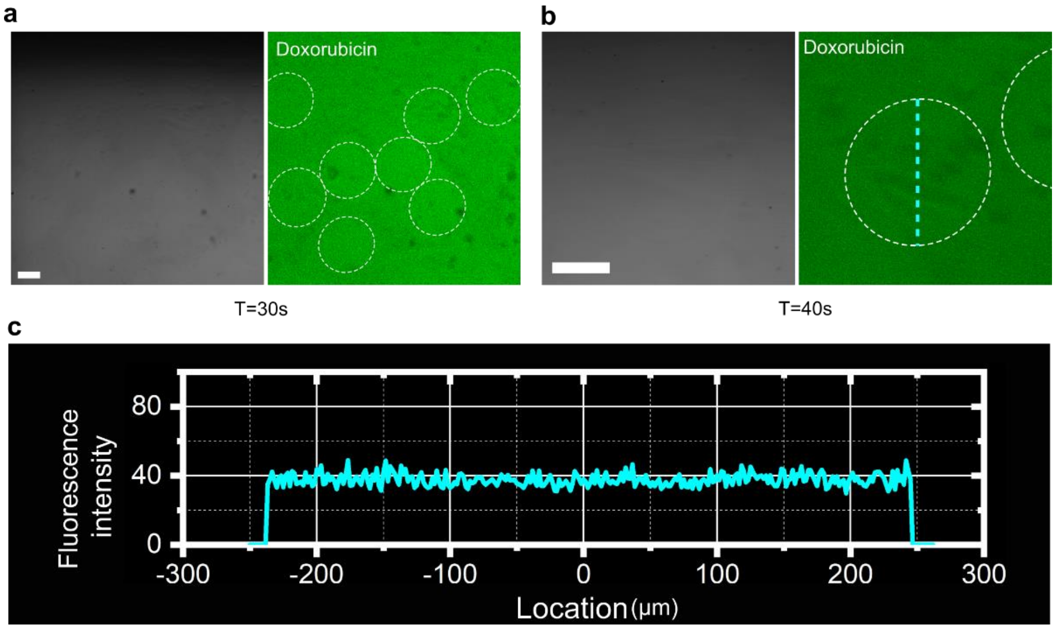

Figure S11 Doxorubicin diffusion in alginate microbeads. (a) Fluorescence image under a 4X objective lens at T=30s. (b) Fluorescence image under a 10X objective lens at T=40s. (c) Fluorescence distribution profile along the diameter. Sale bar:200μm.

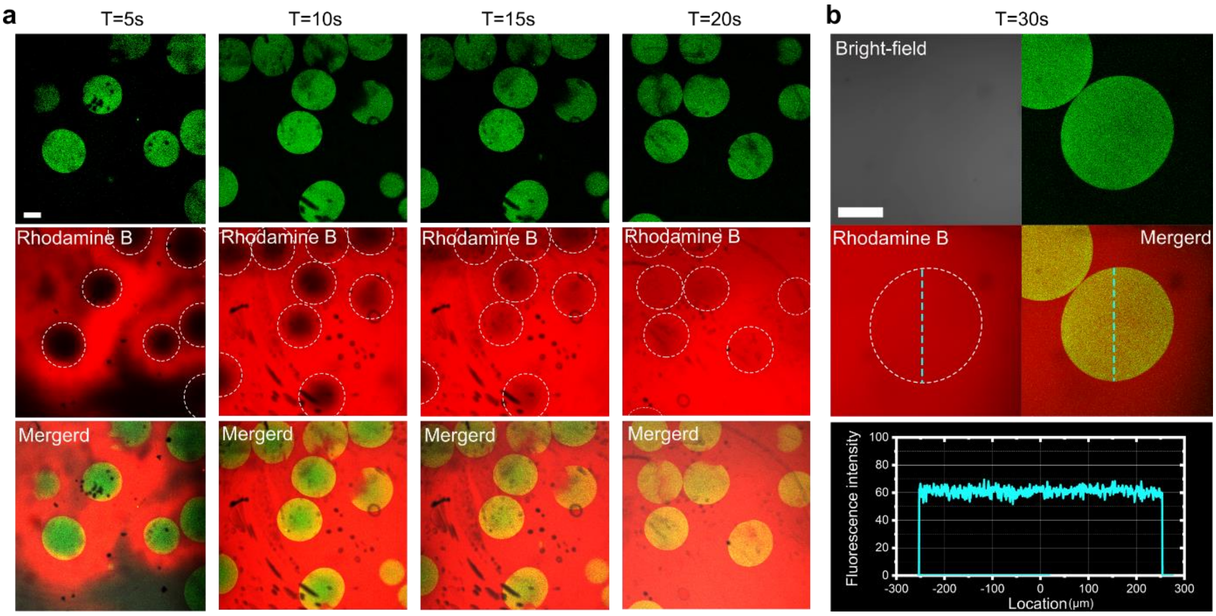

Figure S12 Rhodamine B diffusion in the alginate microbeads. (a) Diffusion process within 20s. (b) Fluorescent images under a 10X objective lens at T=30s and its fluorescent intensity distribution along the diameter. Sable bar: 200 μm.

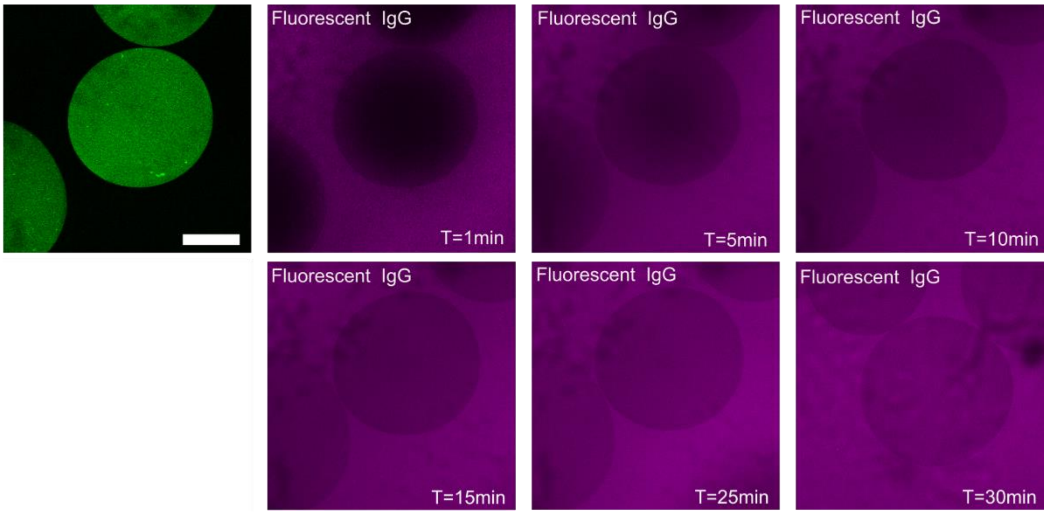

Figure S13. Fluorescent IgG distribution in alginate microbeads. Sable bar: 200 μm.

a b

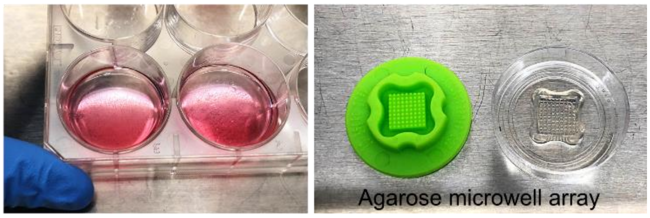

Figure S14. (a) Bulk culture of the alginate microbeads in a six-well culture plate. (b) Agarose microwell array and the commercial mold for the microwell array.
